# Comprehensive analysis of tumour mutational burden and its clinical significance in prostate cancer

**DOI:** 10.1186/s12894-021-00795-7

**Published:** 2021-02-25

**Authors:** Lijuan Wang, Shucheng Pan, Binbin Zhu, Zhenliang Yu, Wei Wang

**Affiliations:** The First Affiliated Hospital, Zhejiang University School of Medicine, Hangzhou, 310003 Zhejiang Province China

**Keywords:** Prostate cancer, Tumour mutational burden, Gene, Clinical

## Abstract

**Background:**

The tumorigenesis of prostate cancer involves genetic mutations. Tumour mutational burden (TMB) is an emerging biomarker for predicting the efficacy of immunotherapy.

**Results:**

Single-nucleotide polymorphisms were the most common variant type, and C>T transversion was the most commonly presented type of single-nucleotide variant. The high-TMB group had lower overall survival (OS) than the low-TMB group. TMB was associated with age, T stage and N stage. Functional enrichment analysis of differentially expressed genes (DEGs) showed that they are involved in pathways related to the terms spindle, chromosomal region, nuclear division, chromosome segregation, cell cycle, oocyte meiosis and other terms associated with DNA mutation and cell proliferation. Six hub genes, PLK1, KIF2C, MELK, EXO1, CEP55 and CDK1, were identified. All the genes were associated with disease-free survival, and CEP55 and CDK1 were associated with OS.

**Conclusions:**

The present study provides a comprehensive analysis of the significance of TMB and DEGs and infiltrating immune cells related to TMB, which provides helpful information for exploring the significance of TMB in prostate cancer.

## Background

Prostate cancer ranks as the second most commonly diagnosed malignancy in males [1]. In terms of treatment, challenges still exist, especially for castration-resistant prostate cancer (CRPC). CRPC with metastasis is reported to have a poor prognosis, with a median survival time of less than 2 years [2]. In recent years, immune checkpoint inhibitors blocking programmed cell death 1 and its ligand (PD-1/PD-L1) and cytotoxic T-lymphocyte antigen-4 (CTLA-4) have shown promising preliminary results in various kinds of tumours. Two clinical trials of ipilimumab in metastatic CRPC have shown improved progression-free survival [3, 4]. Nevertheless, the utilization of immunotherapy is still limited by low efficacy. Some response predictive biomarkers are under investigation, including tumour mutational burden (TMB).

TMB involves the number of non-synonymous somatic mutations per megabase pair (Mbp) of sequenced DNA. Mutations of tumours affect the mutational load, which in turn determines the chance of presenting immunogenically relevant neoantigens [5]. High-TMB tumours tend to harbour more neoantigens than low-TMB tumours, which make the high-TMB tumours more immunogenic, resulting in an improved T cell response and subsequent enhancement of antitumour immunity. Given the function of TMB in immunity, clinical studies focused on melanoma and non-small-cell lung cancer have demonstrated that the TMB is associated with the immunotherapy treatment response [6, 7]. Therefore, TMB is believed to be one of the candidates for predicting the efficacy of immunotherapy.

Next-generation sequencing (NGS) profiling of patients has enabled advancements, and The Cancer Genome Atlas (TCGA) offers convenient access to relevant information. In this study, the gene expression and mutation profiles of prostate cancer samples were extracted from TCGA, and the data were used to investigate the clinical significance of TMB and its related differentially expressed genes (DEGs) and immune cell infiltration signatures.

## Methods

### Data collection and processing

Transcriptome data in the HTSeq‐FPKM format from 524 samples, including 499 prostate and 25 adjacent normal tissue samples, were acquired from the TCGA databank (https://portal.gdc.cancer.gov/). The data were in masked somatic mutation files, and these data were analysed and visualized using the R software package maftools.

### Estimation of TMB and its associations with clinical factors

TMB was defined as the sum of mutations in coding regions per megabase and was calculated as the total number of mutations/the length of exons. The length of exons was estimated to be 38 Mb in previous research [8]. First, Perl scripts were written to obtain the estimated TMB data, which were amalgamated with interrelated survival profiles for each patient. Then, we set the median TMB value as the cutoff, according to which samples were classified into a group with high TMB and a group with low TMB. We utilized the survival R package to analyse the overall survival (OS) differences between the two groups. The differences in TMB between groups categorized based on clinical features (age, T stage and N stage) were analysed using the limma R package.

### Identification of DEGs and DEG pathway analysis

First, we performed DEG analysis with |log_2_ fold change > 1| and false discovery rate (FDR) < 0.05 as cutoffs. We employed the limma package in the R package to analyse the differences and used pheatmap in the R package to generate a heatmap. Subsequently, the gene symbols were transferred into the ID of Entrez using the org.Hs.eg.db package of the R package. Moreover, we employed the ggplot2, enrichplot, and clusterProfiler of R packages to perform Gene Ontology (GO) and Kyoto Encyclopedia of Genes and Genomes (KEGG) analyses of the DEGs. We used GSEA software (https://www.gsea-msigdb.org/gsea/index.jsp) to perform GSEA. We used c2.cp.kegg.v7.0.symbols.gmt as the databank of gene sets. In addition, the TMB level was set as the phenotype labels. The pathways were considered to be statistically enriched according to a cutoff of FDR < 0.25.

### Protein–protein interaction (PPI) network and classification of core genes

We constructed a PPI network of DEGs using STRIING (https://string-db.org/). Evidence-based interactions were generated with a minimum required interaction score > 0.4 [9]. Then, the PPI network was visualized by utilizing Cytoscape 3.8.0 software [10]. Using the CytoHubba plugin, we obtained five protein groups, including the top 30 proteins, with five analytic algorithms, including MCC, MNC, Degree, EPC and EcCentricity, as previously reported [11]. In addition, we acquired the overlapping proteins of the five groups and identified pivotal proteins with more interactions than others as potential hub proteins. These intersections were visualized by a Venn diagram, which was generated online (http://bioinformatics.psb.ugent.be/webtools/Venn/). GEPIA (http://gepia.cancer-pku.cn/) was used to investigate the prognostic significance of the hub proteins.

### CIBERSORT

CIBERSORT is an algorithm for characterizing the immune cell composition of certain tissues according to their gene expression profiles [12]. We used CIBERSORT in the R package to analyse 22 types of immune cells. The comparison of the levels of immune cells between the group with low TMB and the group with high TMB was conducted using the Wilcoxon rank‐sum test, and the results were presented in a violin diagram generated with the vioplot package of the R package.

### Statistical analysis

We used the Kaplan–Meier method to generate the survival curve. The comparisons of OS between different groups categorized according to TMB, age, T stage and N stage was performed using the log‐rank test. We used the Shapiro–Wilk test to determine whether the groups categorized according to TMB, age, T stage and N stage had normal distributions. Through the Shapiro–Wilk test, we found that all the groups were not normally distributed, and thus, nonparametric tests were required. The Wilcoxon rank‐sum test was used to compare groups classified by age and N stage. The Kruskal–Wallis test was used to compare groups classified by T stage. We used R software (version 3.6.1) to perform the Kaplan–Meier analysis, log‐rank test, Wilcoxon rank‐sum test and Kruskal–Wallis test. We used SPSS (version 25.0) to perform the Shapiro–Wilk test. The criterion for statistical significance was a *P* value < 0.05.

## Results

### Mutations in prostate cancer

**Fig. 1:**
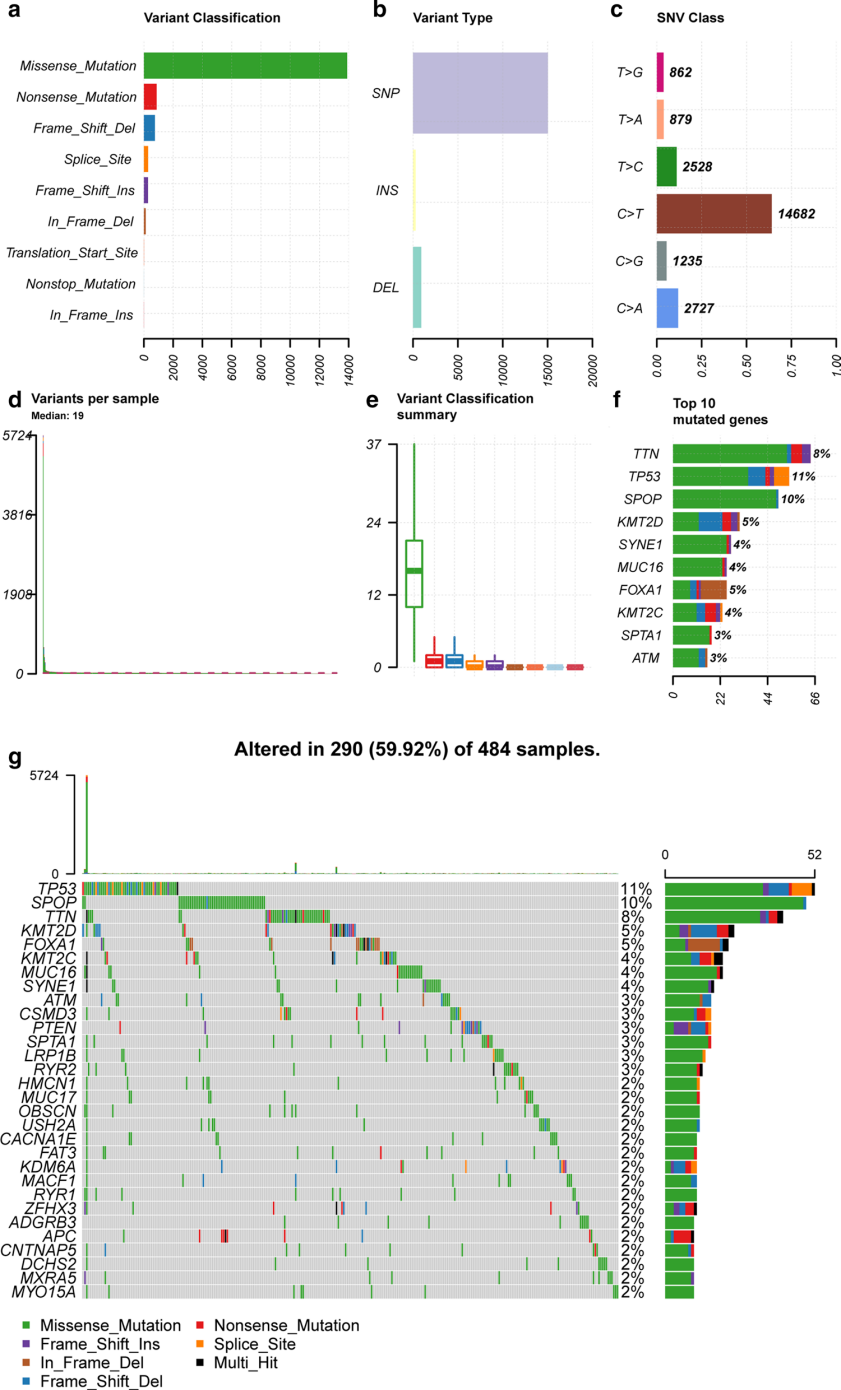
Overview of mutation profile in prostate cancer patients. **a** Variant classification, **b** variant type, **c** SNV class, **d** variants per sample, **e** variant classification summary, **f** top 10 mutated gene, **g** waterfall diagram of top 30 mutated genes

We obtained mutation data from TCGA, which were analysed and visualized with the maftools package. Missense mutations were the most common type of variant (Fig. 1a). The frequency of single-nucleotide polymorphisms (SNPs) was greater than that of other variant types (Fig. 1b). C>T transversions represented the largest proportion of single-nucleotide variants (SNVs) in prostate cancer (Fig. 1c). TTN, TP53, SPOP, KMT2D, SYNE1, MUC16, FOXA1, KMT2C, SPTA1 and ATM were the top ten genes with high mutation frequencies.

### Associations of TMB with prognostic and clinical factors

**Fig. 2:**
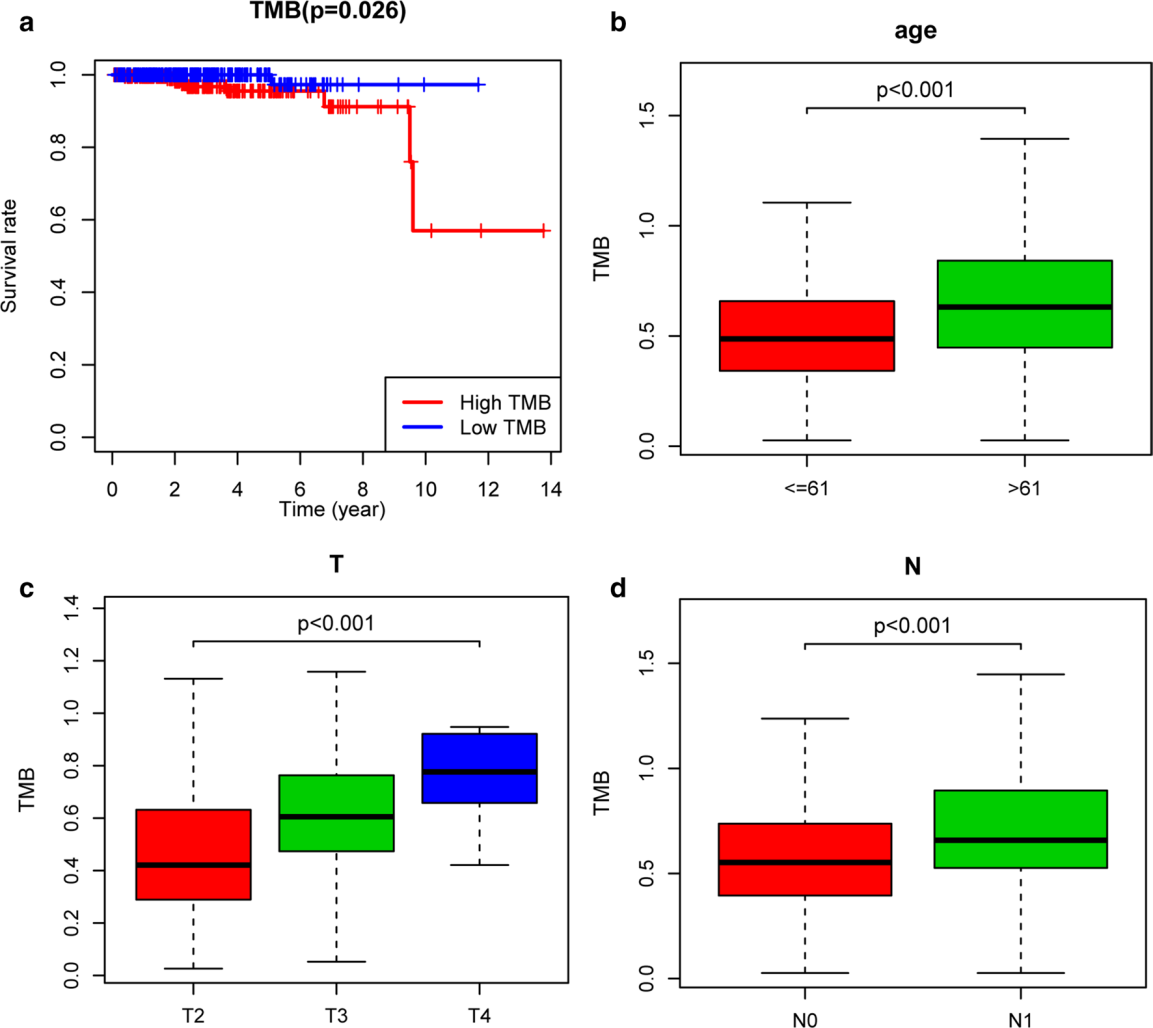
Correlation analysis of TMB with prognostic and clinical factors. **a** Kaplan–Meier curve of OS between high TMB group and low TMB group, **b** the difference of TMB level between groups classified by age, **c** the difference of TMB level between groups classified by T stage, **d** the difference of TMB level between groups classified by N stage

Based on the median TMB, we stratified the patients with prostate cancer into a group with high TMB and a group with low TMB. The prognostic analysis revealed that the group with low TMB had increased OS compared with the group with high TMB (Fig. 2a, *P* = 0.026). There was a relationship between age, T stage, N stage and TMB level (Fig. 2b–d). The median age of the patients was 61 years. It was demonstrated that patients older than 61 years had a higher TMB than those who were 61 years or younger(Fig. 2b, *P* < 0.001). In addition, the higher the T stage was, the higher the TMB (Fig. 2c, *P* < 0.001). Similar results were seen for N stage, with the N1 stage group having a higher TMB than the group with other N stages (Fig. 2d, *P* < 0.001).

### Comparison of DEGs and functional enrichment analysis

**Fig. 3:**
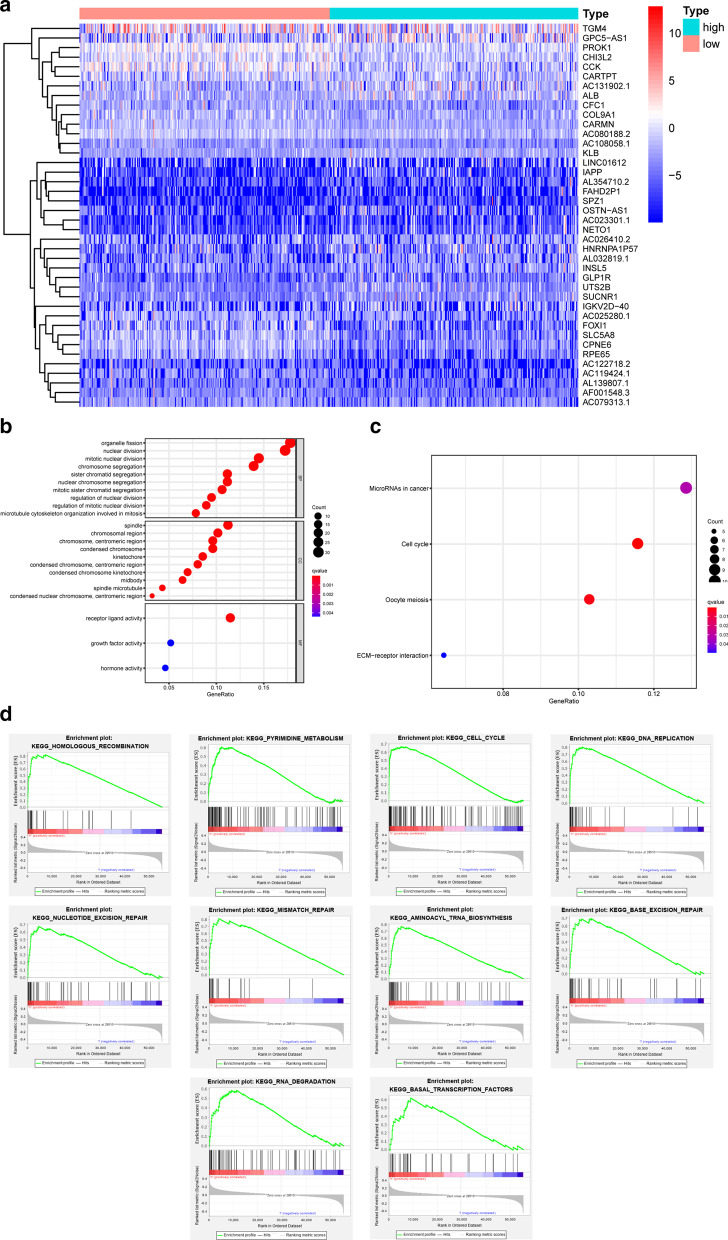
DEGs and its corresponding enrichment analysis. **a** Heatmap of the top 20 DEGs, **b** GO enrichment of the DEGs, **c** KEGG enrichment of the DEGs, **d** top 10 pathways of the GSEA enrichment

A total of 257 DEGs were identified, and the top 20 DEGs were displayed in the heatmap (Fig. 3a). Functional enrichment analysis of the DEGs was conducted. GO enrichment analysis, including three major categories, was utilized (Fig. 3b). In the biological process (BP) category, the terms nuclear division, organelle fission and chromosome segregation were enriched. The cellular component (CC) category terms included spindle, chromosomal region, kinetochore, midbody and microtubule. The molecular function (MF) category terms involved receptor-ligand activity, growth factor activity and hormone activity. Moreover, we identified pathways related to the terms microRNA in cancer, cell cycle, oocyte meiosis and ECM-receptor interaction in the KEGG enrichment analysis (Fig. 3c). There were 37 pathways enriched in the GSEA, and the top 10 pathways were displayed in a diagram (Fig. 3d). These pathways were related to DNA-level cell proliferation, including mechanisms such as pyrimidine metabolism, DNA replication, DNA degradation, and aminoacyl tRNA biosynthesis, and the findings were in accordance with the above results.

### PPI network of DEGs and selected hub genes

**Fig. 4:**
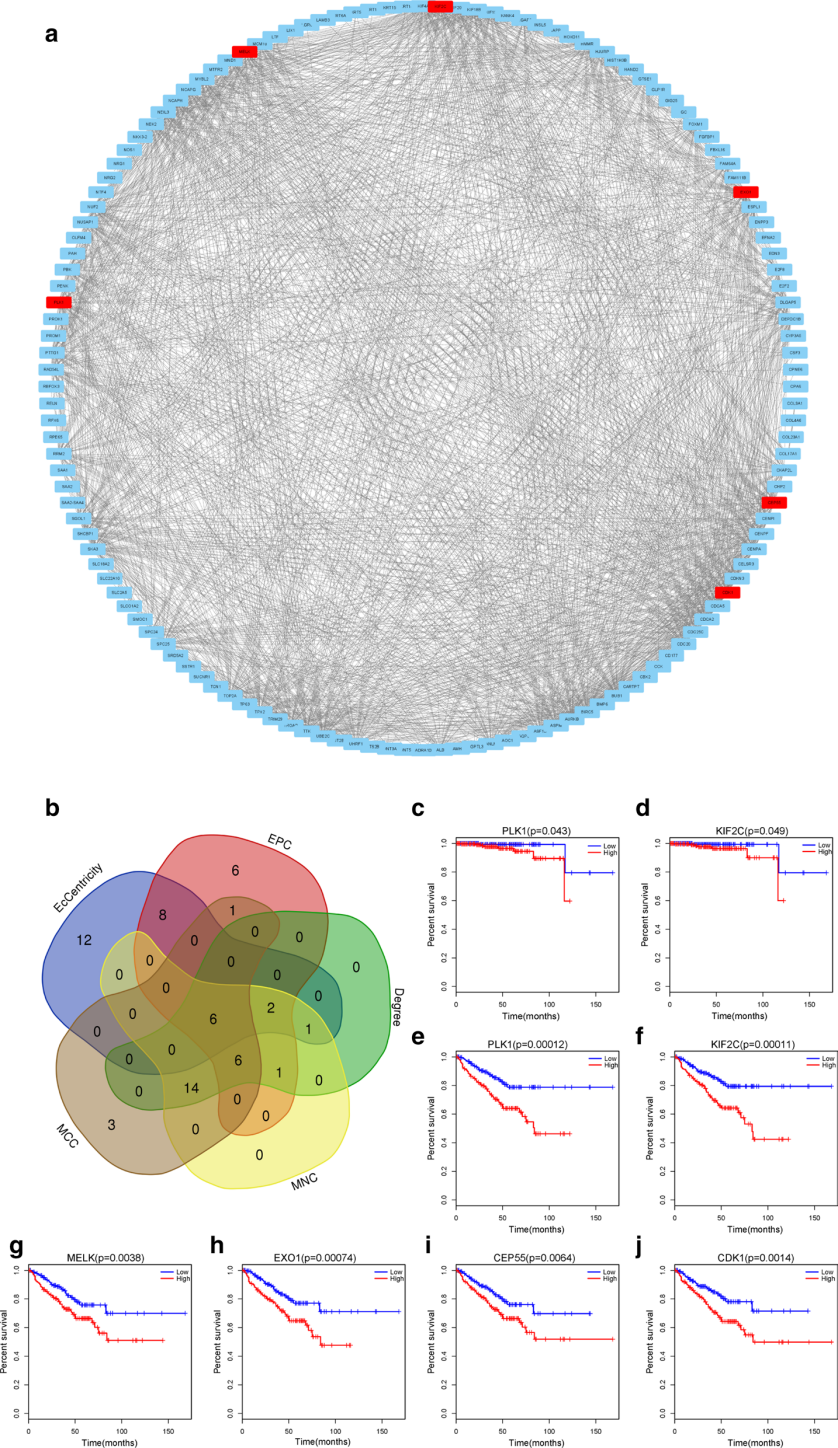
PPI network and hub genes with their correlation to survival. **a** PPI network of DEGs generated by Cytoscape with hub genes showing red color, **b** Venn dots of intersections from five methodology involved MCC, MNC, Degree, EPC and EcCentricity, **c**–**j** Logrank analysis of hubgenes. The relation of PLK1 (**c**) and KIF2C (**d**) with overall survival in prostate cancer patients. The relation of PLK (**e**), KIF2C (**f**), MELK (**g**), EXO1 (**h**), CEP55 (**i**) and CDK1 (**j**) with disease free survival in prostate cancer patients

A PPI network of 189 nodes and 1789 edges was generated by STRING, and online tool for analysing proteins, and the results were visualized by Cytoscape (Fig. 4a). Five algorithms, MCC, MNC, Degree, EPC and EcCentricity, were utilized, and the overlapping proteins in the results generated from each algorithm were identified with Venn diagrams (Fig. 4b). PLK1, KIF2C, MELK, EXO1, CEP55 and CDK1 were identified as hub genes through this method. High expression of PLK1 and KIF2C was related to poor overall survival, with a *P* value < 0.05. High expression of PLK1, KIF2C, MELK, EXO1, CEP55 and CDK1 was related to poor disease-free survival, with a *P* value < 0.05.

### Comparison of differential immune cell signatures

**Fig. 5:**
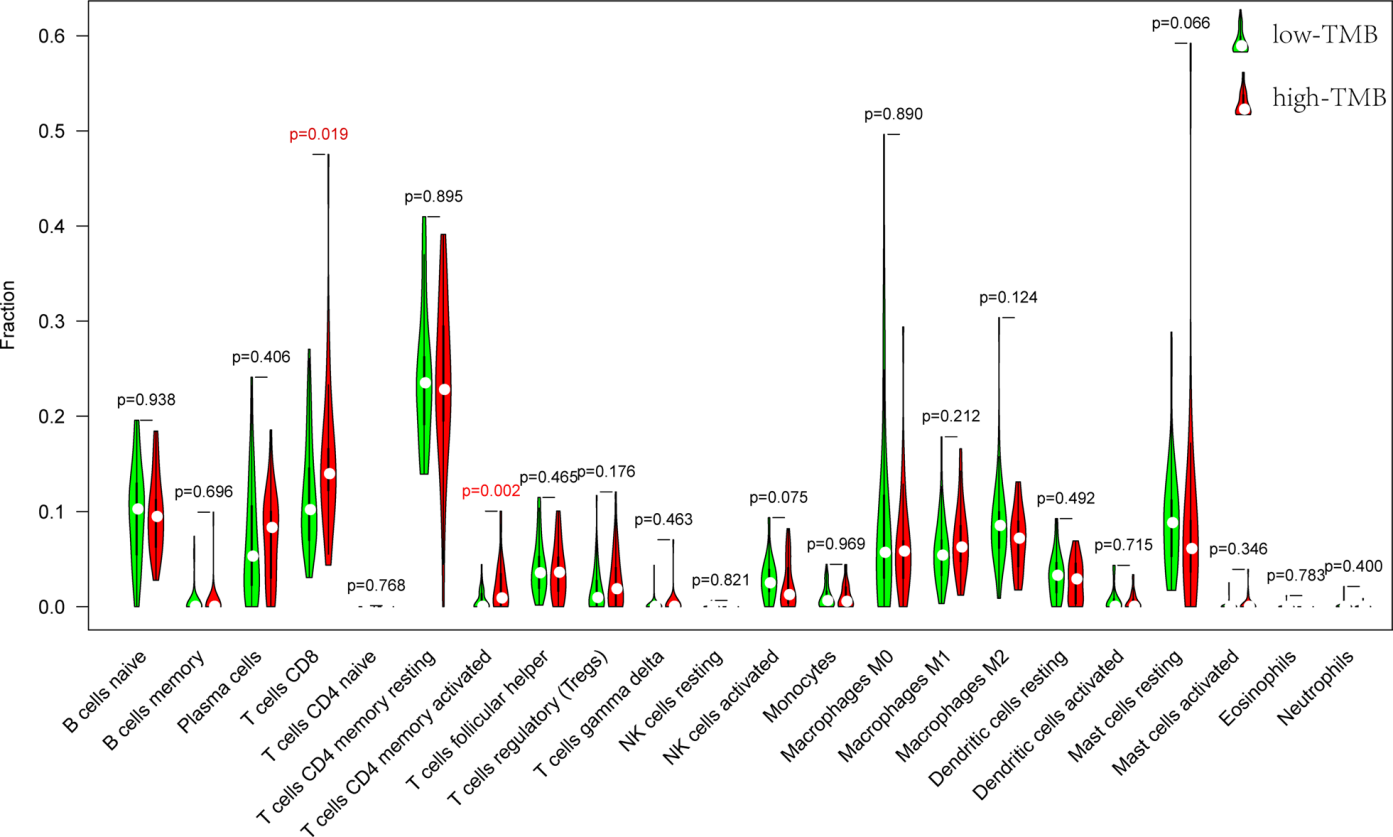
Comparisons of 22 immune cell infiltrations between low‐TMB groups and high‐TMB groups

A violin diagram was generated to visualize the differences in the proportions of 22 infiltrating immune cells between the group with low TMB and the group with high TMB (Fig. 5). The group with high TMB had higher levels of CD8 T cells and activated CD4 memory cells than the low TMB group (*P* < 0.05). However, there were no other statistically significant findings or trends for the other infiltrating immune cells.

## Discussion

Immunotherapies have shown preliminary results in prostate cancer. TMB is considered an emerging biomarker for response evaluation. Therefore, it is meaningful to investigate the relationship between TMB and prostate cancer. This study provides an overview of mutations, the clinical significance of TMB, and DEGs and infiltrating immune cells related to TMB in prostate cancer.

Among the top 10 mutated genes, TP53, SPOP, FOXA1 and ATM showed pivotal functions in the initiation and development of malignant prostate cancer. As a tumour suppressor, TP53 has a high mutation frequency among various kinds of tumours, and the mutant form is equipped with antiproliferative functions and is related to the metastasis and progression of prostate cancer [13, 14]. Mutations in SPOP are considered the most common recurrent point mutations in prostate cancer [15]. SPOP is crucial for the preservation of nuclear genome stability and is essential for the degradation of multiple proteins [16, 17]. FOXA1 is necessary for androgen receptor-mediated activation of prostate genes [18]. Furthermore, the expression of FOXA1 is related to tumorigenesis and the progression of prostate cancer [19]. ATM is considered one of the DNA damage repair genes, and its activation can be seen in the earlier stages of prostate tumorigenesis [20].

The clinical significance of TMB in prostate cancer was assessed. Similar to a previous prostate and renal cancer study, our study found that the group of patients with prostate cancer with high TMB had lower OS than the group of patients with prostate cancer with low TMB^[1]^[21]. Furthermore, we observed higher TMB levels in patients older than 61 years than in those who were 61 years or younger and in the higher T stage and N1 stage groups. According to reports from the TCGA database, the prognostic role of TMB is unclear. In a study of bladder cancer, the high TMB group exhibited increased OS compared with the low TMB group [22]. Two reasons probably account for the results of our research. One reason is that not all patients with a high TMB have an increased treatment response, as not every generated neoantigen has immunogenicity [23]. That is, high TMB does not always initiate an antitumour response. Another reason is that the high TMB group in this study was older and had a more advanced stage than the low TMB group.

GO and KEGG analyses revealed that the DEGs between the group with high TMB and the group with low TMB were related to the terms spindle, chromosomal region, kinetochore, nuclear division, chromosome segregation, cell cycle, oocyte meiosis, receptor-ligand activity, growth factor activity and ECM-receptor interaction, all of which are associated with DNA mutation and cell proliferation. Furthermore, the results of GSEA also supported this observation, showing enrichment of pathways related to pyrimidine metabolism, DNA replication, DNA degradation and aminoacyl tRNA biosynthesis. None of the analyses showed associations with pathways related to immune mediation or response. This phenomenon is likely because the immunogenicity of prostate cancer cases with low TMB is poorer than that of lung cancer and melanoma [24]. Because of this poor immunogenicity, the numbers of neoantigens generated by prostate cancer patients may be less than those generated by high-TMB cancer patients. The subsequent immune response in patients with low TMB is probably also weaker than that of high-TMB cancer patients.

Through CytoHubba analysis of the PPI network, we identified six hub genes: PLK1, KIF2C, MELK, EXO1, CEP55 and CDK1. All the genes were correlated with DFS, and CEP55 and CDK1 were associated with OS. PLK1 and MELK have been suggested to be potential targets in prostate cancer. As PLK1 plays a critical role in the proliferation of cells, centrosome abnormalities, mediation of the cell cycle and apoptosis, it is considered a potential treatment target in prostate cancer [25]. It has been reported that targeting PLK1 can enhance the response to androgen signalling inhibitors or olaparib in CRPC [26, 27]. MELK is upregulated in prostate cancer and related to aggressiveness. Furthermore, in vitro silencing of MELK can weaken the proliferation of prostate cancer cells, and in vivo tests also proved that an inhibitor of MELK could repress the growth of prostate cancer.

Different kinds of immune cells play a pivotal role in the tumour microenvironment and are also determinants of immunotherapy efficacy. Therefore, our study explored differences in the levels of immune cells between the group with high TMB and the group with low TMB. The analysis indicated that the group with high TMB had a significantly higher proportion of CD8 T cells and activated CD4 memory cells than the group with low TMB. These findings were similar to those in previous reports of bladder cancer [22]. Generally, a high TMB can produce more neoantigens, eliciting a subsequent immune response. CD8 T cells are one of the determinants of antigen-specific responses. Therefore, the high TMB group with higher levels of CD8 T cells is likely to experience superior immunotherapy efficacy. However, a recent study demonstrated that metastatic CRPC with low TMB but a high density of CD8 T cells could also benefit from immune checkpoint inhibitors [28]. Our findings suggesting that TMB has an interaction with immune infiltration are only preliminary, and further validation in clinical cohorts and investigations to explore the underlying mechanisms of the correlations are needed.

The study has some limitations that should be considered. Firstly, all the data were retrospectively collected which had bias. Secondly, the results of our study were preliminary exploration and should be further tested through in vitro or in vivo experiments. Thirdly, our study lacked sub-group analysis including CRPC patients and non-CRPC patients.

## Conclusion

In conclusion, we performed a comprehensive and systematic analysis of TMB in prostate cancer and analysed its clinical significance. Furthermore, we also identified enriched pathways of DEGs, hub genes with prognostic roles and infiltrating immune cells related to TMB. Our results elucidate the association between TMB and infiltrating immune cells in prostate malignancy and will likely be useful for future investigations of TMB in prostate cancer.

## Supplementary Information

**Additional file 1.** **Table S1.** Setting items of the transcriptome data. **Table S2.** Setting items of the clinical data. **Table S3.** Setting items of the mutation data.

## Data Availability

The datasets used and/or analyzed during the current study are available from the TCGA databank (https://portal.gdc.cancer.gov/). Transcriptome data, clinical data, and mutation data could be downloaded according to the supplementary file's detailed setting items (Additional file 1: Table S1, Table S2 and Table S3).

## Abbreviations

TMB: Tumour mutational burden
SNPs: Single nucleotide polymorphisms
SNV: Single-nucleotide variant
OS: Overall survival
DFS: Disease free survival
CRPC: Castration-resistant prostate cancer
PD-1/PD-L1: Programmed cell death 1 and its ligand
CTLA-4: Cytotoxic T-lymphocyte antigen-4
Mbp: Megabase pair
NGS: Next-generation sequencing
TCGA: The Cancer Genome Atlas
GO: Gene Ontology
KEGG: Kyoto Encyclopedia of Genes and Genomes
PPI: Protein–protein interaction
BP: Biological process
CC: Cellular component
MF: Molecular function
