# Supplementary material for: Comprehensive analysis of tumour mutational burden and its clinical significance in prostate cancer
Source: BMC Urol. 2021 Feb 25;21:29. doi: 10.1186/s12894-021-00795-7 (PMC7905899; doi:10.1186/s12894-021-00795-7)
Supplement: Supplementary file 1 — Additional file 1. Table S1. Setting items of the transcriptome data. Table S2. Setting items of the clinical data. Table S3. Setting items of the mutation data. [file 12894_2021_795_MOESM1_ESM.docx]

Detailed setting items of the data

Open the TCGA databank (<https://portal.gdc.cancer.gov/>). The setting items of transcriptome data could accord to items in table1. When finishing all the items, click the button of “add all files to chart”. Open the chart and click the button of download. The setting items of clinical data could accord to items in table2. When finishing all the items, click the button of “add all files to chart”. Open the chart and click the button of download. The setting items of mutation data could accord to items in table3. When finishing all the items, click the button of “add all files to chart”. Open the chart and click the button of download.

Table S1 Setting items of the transcriptome data

| Files | | Cases | |
| --- | --- | --- | --- |
| Data Category | transcriptome profiling | Primary Site | prostate gland |
| Data type | Gene Expression Quantification | Program | TCGA |
| Workflow Type | HTSeq - FPKM | Project | TCGA-PRAD |
| Data Format | txt |  |  |

Table S2 Setting items of the clinical data

| Files | | Cases | |
| --- | --- | --- | --- |
| Data Category | Clinical | Primary Site | prostate gland |
| Data type | Clinical Supplement | Program | TCGA |
| Data Format | bcr xml | Project | TCGA-PRAD |

Table S3 Setting items of the mutation data

| Files | | Cases | |
| --- | --- | --- | --- |
| Data Category | simple nucleotide variation | Primary Site | prostate gland |
| Data type | Masked Somatic Mutation | Program | TCGA |
| Workflow Type | VarScan2 Variant Aggregation and Masking | Project | TCGA-PRAD |
| Data Format | maf |  |  |
